# Proteomics Analysis of the Effects of Cyanate on *Chromobacterium violaceum* Metabolism

**DOI:** 10.3390/genes2040736

**Published:** 2011-10-19

**Authors:** Rafael A. Baraúna, Alessandra Ciprandi, Agenor V. Santos, Marta S.P. Carepo, Evonnildo C. Gonçalves, Maria P.C. Schneider, Artur Silva

**Affiliations:** 1 Laboratório de Polimorfismo de DNA, Instituto de Ciências Biológicas, Universidade Federal do Pará, Belém, Pará 66075-110, Brasil; E-Mails: rabarauna@ufpa.br (R.A.B.); alecip@gmail.com (A.C.); agenorvaladares@yahoo.com.br (A.V.S.); ecostag@ufpa.br (E.C.G.); paula@ufpa.br (M.P.C.S.); 2 REQUIMTE/CQFB, Departamento de Química, Faculdade de Ciências e Tecnologia, Universidade Nova de Lisboa, Caparica 2829-516, Portugal; E-Mail: marta.carepo@dq.fct.unl.pt (M.S.P.C.)

**Keywords:** 2DE, bacteria, cyanate, mass spectrometry, proteomic

## Abstract

*Chromobacterium violaceum* is a gram-negative betaproteobacterium that has been isolated from various Brazilian ecosystems. Its genome contains the cyn operon, which gives it the ability to metabolize highly toxic cyanate into ammonium and carbon dioxide. We used a proteomics approach to investigate the effects of cyanate on the metabolism of this bacterium. The proteome of cells grown with and without cyanate was compared on 2-D gels. Differential spots were digested and identified by mass spectrometry. The bacterium was able to grow at concentrations of up to 1 mM cyanate. Eighteen spots were differentially expressed in the presence of cyanate, of which 16 were downregulated and only two were upregulated. An additional 12 spots were detected only in extracts of cells unexposed to cyanate, and one was expressed only by the exposed cells. Fourteen spots were identified, corresponding to 13 different proteins. We conclude that cyanate promotes expression of enzymes that combat oxidative stress and represses enzymes of the citric acid cycle, strongly affecting the energetic metabolism of the cell. Other proteins that were under-expressed in bacteria exposed to cyanate are involved in amino-acid metabolism or are hypothetical proteins, demonstrating that cyanate also affects expression of genes that are not part of the cyn operon.

## 1. Introduction

*Chromobacterium violaceum* is a free-living gram-negative betaproteobacterium that has been isolated from various tropical and subtropical regions, including the Amazon forest [1,2], the Brazilian Cerrado (savanna) and the Atlantic rain forest [3]. This species has a very versatile metabolism, and is thus able to survive in a variety of different environments [4]. The genome of strain ATCC 12472, isolated from a freshwater environment in Mentakab, Malaysia, has been sequenced [5]. This strain has biochemical and molecular characteristics similar to those of strains isolated from the Amazon region and the Atlantic rain forest in Brazil [3]. A number of genes organized as operons and involved in detoxification of environmental pollutants were identified in *C. violaceum* ATCC 12472 [5,6], including the *cynRST* operon, which determines resistance to cyanate (CNO^−^). Bacteria with this operon are able to metabolize cyanate into ammonia and carbon dioxide, which are then used in cellular metabolism [6,7].

Cyanate is produced in the cell as an intermediate product in the biosynthesis of amino acids and in nature through the spontaneous dissociation of urea, a process that has been known for some time [8]. This compound is also a component of the chemical waste produced during the recovery of gold and other metals from mines, due to photo-oxidation of cyanide (CN^−^) discharged into mine-waste impoundments [9]. The highly toxic nature of cyanate for living organisms has been well documented [10,11]. For many bacteria, however, this compound can serve as a nitrogen source [12,13].

Although the functional mechanisms of the *cyn* operon are well known [7,13,14], the influence of cyanate on the expression of other genes has not been investigated. These genes may play fundamental roles in the processes of cyanate assimilation and degradation, and in the subsequent reduction of this pollutant in the environment. Proteomic approaches are widely-used for the identification of differentially-expressed proteins, through techniques such as two-dimensional electrophoresis (2-D), coupled with mass spectrometry [15-18].

Knowledge concerning the effect of cyanate on bacterial metabolism is crucial for understanding how they can eliminate this pollutant. We exposed *C. violaceum* to cyanate, and obtained protein extracts from exposed and unexposed bacteria for characterization of the proteome associated with exposure to this compound.

## 2. Results and Discussion

### 2.1. Bacterial Resistance to Cyanate

**Figure 1:**
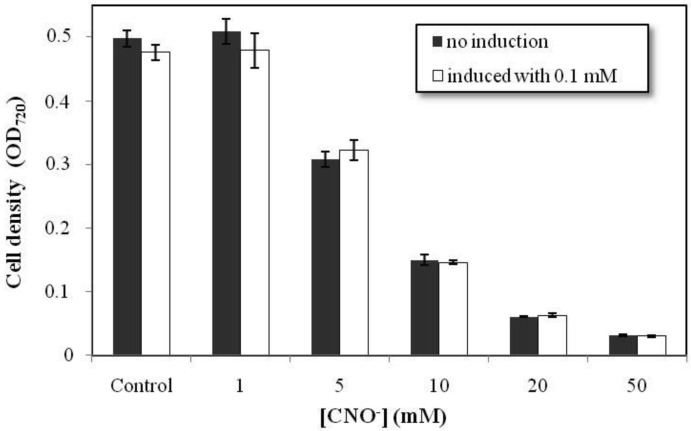
Resistance of *Chromobacterium violaceum* to cyanate (CNO^−^). The resistance assays were conducted at five concentrations of cyanate (1, 5, 10, 20 and 50 mM), using two groups of cells, not induced and induced with 0.1 mM cyanate. The error bars indicate the standard deviations for the mean values derived from the analyses in triplicate. Data on the growth of the two groups was compared using ANOVA, with a *p* < 0.05 significance level.

*Chromobacterium violaceum* was grown in various concentrations of cyanate in order to evaluate its resistance to this compound. The bacteria grow well at concentrations of cyanate of up to 1 mM (Figure 1). At 5 mM, growth was 67% of that observed in the control group. Thus, *C. violaceum* was able to grow in concentrations of cyanate normally founded in aquatic environments associated with mine tailings [19]. Above 10 mM, however, *C. violaceum* was unable to metabolize the cyanate effectively, and growth was inhibited considerably. At 50 mM, the bacterial growth was close to zero. Resistance tests were conducted on two groups of bacteria, one of which was initially cultured in medium with a low concentration of cyanate (0.1 mM) prior to exposure to higher experimental concentrations (white bars in Figure 1). This procedure was used to test whether exposure to small doses of this toxic compound would increase the resistance of the bacteria. However, no significant difference in resistance was found between the groups (Figure 1). This result indicates that probably the *cyn* operon is not responsible for the *C. violaceum* resistance to cyanate. The ability of *Escherichia coli* and bacteria of the genus *Pseudomonas* to grow in the presence of cyanate has been described but the *cyn* operon is not always involved in the resistance [13,20,21]. In *Pseudomonas pseudoalcaligenes* the tolerance to cyanate of a *cyn*S mutant was similar to the wild type showing that the *cyn* operon is not involved in the resistance mechanism [13].

### 2.2. Comparative Proteomics

**Figure 2:**
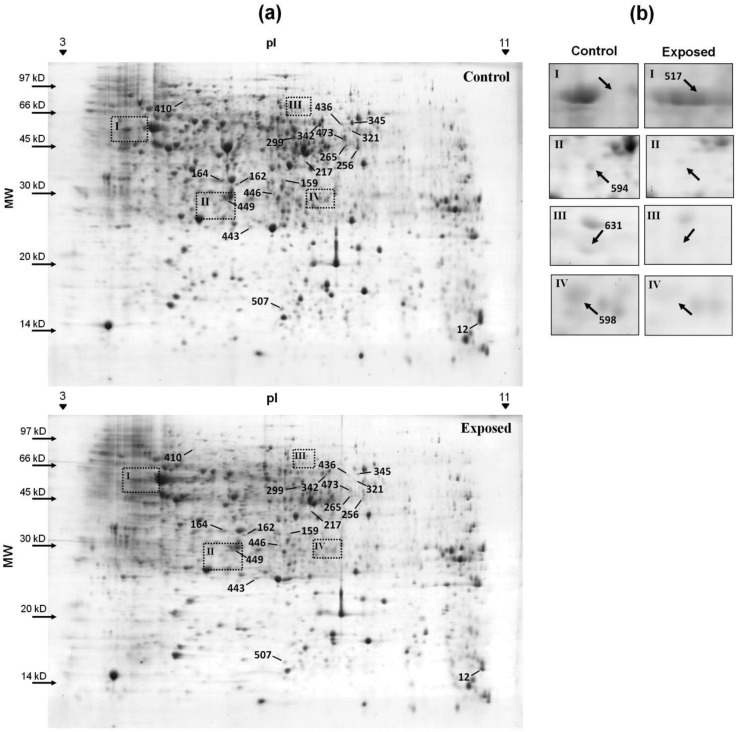
(**a**) Two-dimensional gels of the soluble proteins of *Chromobacterium violaceum*. The 18 differential spots are indicated and identified by their match ID. Spots 443 and 449 were upregulated, while all the other were downregulated. Molecular weight is shown on the left, and the pH range of the strip used in the first dimension is shown in the upper part of the gel. The comparative analysis was conducted with three biological replicates; (**b**) A detailed view of the spots detected under one of the conditions.

To measure the changes in protein expression when cyanate was added to the growth medium, the protein profiles of cells grown with and without cyanate were compared in 2-D gels. The bacteria were exposed to a concentration of 1 mM cyanate. Over 71% of the spots of each gel coincided among the three biological replicates (data not shown). Comparative analysis demonstrated that expression of 18 proteins was affected by cyanate in the culture medium. Of these, 16 were downregulated; only two were upregulated (Figure 2a). An additional 12 spots were detected exclusively in the control group, and one was only found in the exposed group (Figure 2b). Fourteen spots, corresponding to 13 different proteins, were identified using mass spectrometry (Table 1) (see supplementary material for more information of the identified spots). All of these proteins were classified as cytoplasmatic, with the exception of superoxide dismutase, which was characterized as extracellular by PSLpred [22] and periplasmatic by the PSORTb v.3.0 program [23]. All of the differently expressed proteins had a theoretical pI of between 5 and 7. Only the 30S ribosomal protein S10 had an alkaline pI of 9.62.

No proteins encoded by the *cyn* operon were identified with differential expression. This result may indicate that these proteins are in low concentration in comparison with other proteins of the cellular metabolism, making it difficult its detection in the gel, or are among the differentially expressed proteins that could not be identified by MS/MS. A third explanation is that there is no differential expression of these proteins under the tested conditions. This could explain the fact that there is no increase in the bacteria resistance when the culture is pre-induced with cyanate.

**Table 1:** Proteins that were differentially expressed by *Chromobacterium violaceum* due to exposure to cyanate, identified in a mass spectrograph. Proteins are organized in the table according to COGs (Clusters of Orthologous Groups of Proteins). The relative volumes with a minus sign represent proteins that were downregulated, while those without a sign were upregulated.

**Match ID**	**Mascot score**	**Accession number**	**Protein name**	**Relative volume**	**Theoretical pI/MW**
***Energy production and conversion***					
299	63	34496527	Dihydrolipoamide acetyltransferase	−4.10007	6.28 / 43031
345	108	34102383	Dihydrolipoamide dehydrogenase	−2.26577	6.55 / 50020
***Inorganic ion transport and metabolism***					
443	202	34103814	Superoxide dismutase	1.40629	5.87 / 21634
***Translation, ribosomal structure and biogenesis***					
12	164	34105492	30S ribosomal protein S10	−1.52632	9.62 / 11696
164	113	34499644	Elongation factor G	−1.84014	5.23 / 77244
631	134	34105448	hoxX-like protein	0 *	6.02 / 63225
***Amino acid transport and metabolism***					
342	343	46576431	Serine hydroxymethyltransferase	−2.82757	6.24 / 45060
159	161	34104883	Dihydrodipicolinate synthase	−2.12351	5.88 / 30526
***Post translational modification, protein turnover, chaperones***					
594	80	34105312	Stringent starvation protein A	0 *	5.77 / 23224
***Lipid metabolism***					
598	79	34103391	3-hydroxyisobutyrate dehydrogenase	0 *	6.24 / 30145
***Carbohydrate transport and metabolism***					
517	52	34104763	Phosphopyruvate hydratase	0 **	5.14 / 22400
***Function unknown***					
162	90	34105156	Hypothetical protein	−1.3472	5.68 / 32309
***Unrelated COG***					
507	133	34104388	Hypothetical protein	−2.09969	5.30 / 12987

* Protein detected only in the control group;

** Protein detected only in the exposed group.

An *in silico* predicted proteome was also produced using the online program JVirGel v.2.0 [24] (supplementary material). This predicted proteome had the same pattern as the experimental gel in the acidic pH range; few proteins were visualized at pH values close to 3. However, in the alkaline range the *in silico* prediction indicated a much larger number of spots than that observed in the gel pattern.

The proteins whose expression was affected by cyanate were classified into COG classes C, E, G, I, J, M, O, P, and S (Table 1). One of the spots identified as a hypothetical protein (CV_3077) was not related to any COG. Proteomic approaches have been used to characterize and validate hypothetical proteins [25,26]. In our study, two hypothetical proteins were described and thus had their genomic prediction confirmed. These proteins were among those under-expressed in the presence of cyanate; they are encoded by the CV_3077 (Match ID 507) and CV_3850 (Match ID 162) loci. The YicC domain was detected by InterProScan [27] in the *N*-terminal portion of the hypothetical protein of the CV_3850 locus. This protein domain has been observed in proteins responsible for bacterial survival during the stationary phase of cell growth and during periods of stress due to high temperatures [28]. We found that this protein domain is expressed during the exponential growth phase in *C. violaceum*, and that cyanate reduces its expression. The other hypothetical protein (CV_3077 locus) has 177 amino acids, and has a NAD(P) binding domain in the *N*-terminal portion of the chain.

Superoxide dismutase enzyme (SOD) was one of the over-expressed proteins, expressed at a rate 1.4 times greater than in the control. We were not able to identify the other over-expressed protein. Increased expression of SOD, which is responsible for dismutation of superoxide radicals, suggests that cyanate induces oxidative stress in *C. violaceum*, a scenario normally associated with senescence [29]. Other chemical compounds, such as arsenic, can also induce oxidative stress in bacterial cells, promoting over-expression of the SOD enzyme [30]. Superoxide radicals preferentially attack specific groups of proteins [31], such as dihydrolipoamide acetyltransferase and dihydrolipoamide dehydrogenase, which were among the least expressed proteins in our study. These enzymes are components of the multi-enzymatic 2-oxoglutarate dehydrogenase complex and are involved in the production of energy by the citric acid cycle. Dihydrolipoamide acetyltransferase was the protein most affected by cyanate, with a spot volume 4.1 times smaller in the extracts from bacteria treated with cyanate.

This indicates that, under conditions of chemical stress, such as exposure to cyanate, this bacterium uses mechanisms similar to those employed for the prolongation of their lifespan during the stationary growth phase, inducing oxidative stress proteins and repressing aerobic respiratory cycle enzymes [31].

The enzymes dihydrodipicolinate synthetase and serine hydroxymethyltransferase, which are involved in amino-acid synthesis, were also downregulated (Table 1). The latter enzyme catalyzes interconversion of the amino acid glycine into serine. Conversion of glycine into cyanide by bacteria of the genus *Chromobacterium* is well documented [32,33]. The genome of *C. violaceum* ATCC 12472 contains the *hcnBCA* genes responsible for this conversion [6]. Cyanide may also be formed through the spontaneous dissolution of cyanate [19]. Thus, high concentrations of glycine and cyanate in the environment can contribute to intracellular production of cyanide, which is extremely toxic at high concentrations. Repression of serine hydroxymethyltransferase, which converts serine into glycine, may be a mechanism to control concentrations of this amino acid, which is maintained at basal levels, allowing the cell to preferentially use the cyanide obtained from the dissolution of cyanate. Serine hydroxymethyltransferase was identified in two spots of the proteome (342 and 473), with a difference of 8 kD in molecular mass. This difference can be attributed to post-translational modifications.

The other under-expressed proteins are involved in the translation and structure of ribosomes (Table 1); they are found in many differential proteomes [34,35]. Given that these proteins are so commonly found under stress conditions leads to speculation about whether they are a part a cellular response to stress or are simply technical artifacts [34,35].

Twelve proteins were only visualized in the gels of the control group; three of these were identified (Figure 2b). Stringent starvation protein A (SspA) is an RNA-polymerase-associated protein that is essential for lytic growth of the P1 bacteriophage in *E. coli* [36]. This protein has also been shown to be essential for the survival of the cell when nitrogen is scarce [37]. As cyanate is a rich source of nitrogen, SspA may no longer be essential for the cell, and is thus completely repressed. The other two proteins that were identified were HoxX and the enzyme 3-hydroxy-isobutyrate dehydrogenase; the latter is involved in degradation of the amino acid valine. The protein HoxX is expressed in conjunction with HoxA for transcriptional regulation of genes coding for dehydrogenase enzymes [38,39]. The *hoxA* gene is absent in *C. violaceum*; the functional interactions predicted by the STRING v.8.3 program [40] for the *hoxX* gene revealed its interaction with uncharacterized proteins or transcriptional regulators.

The protein detected only in the presence of cyanate was the enzyme phosphopyruvate hydratase, which is involved in the final step of the conversion of glucose into pyruvate. As the enzymes involved in energy production by the citric acid cycle are repressed and *C. violaceum* is able to grow under both aerobic and anaerobic conditions [41], the pyruvate formed in glycolysis is probably used for generation of energy through the production of acetic and formic acid. This pathway is normally employed to obtain energy only under anaerobic conditions. However stress conditions caused by cyanate may activate this pathway.

## 3. Experimental Section

### 3.1. Bacterial Culture Conditions

The samples of *C. violaceum* ATCC 12472 were stored in 25% glycerol at −70 °C. The bacteria were cultivated in LB medium at a constant temperature of 28 °C and 180 rpm agitation. Growth was monitored by measuring 720 nm optical density (OD_720_), since the pigment violacein produced by the bacteria interferes with optical density readings at 600 nm. Potassium cyanate (KCNO) (Sigma-Aldrich) was employed as a source of the cyanate ion for the exposure assays.

### 3.2. Resistance Analysis

The bacteria were initially inoculated in LB agar at 28 °C. Two colonies were selected from the Petri dish and inoculated into two tubes with liquid LB, one of which contained 0.1 mM potassium cyanate. These bacteria were incubated overnight at 28 °C and 180 rpm agitation. Subsequently, each culture was diluted to an OD720 of 0.06, which corresponds to the beginning of the log phase; the LB liquid had cyanate concentrations that varied from 1 to 50 mM. A control group without cyanate was also prepared. These media were again incubated at 28 °C and 180 rpm until the mid-log phase, and the absorbance was measured in an Ultrospec 5300 *pro* spectrophotometer (Amersham Biosciences). All cyanate concentrations were tested in triplicate. The highest concentration of cyanate at which there was no inhibition of growth was used for proteomic analysis.

### 3.3. Protein Extraction and Quantification

For protein extraction, 100 mL aliquots of bacterial cultures exposed or unexposed to cyanate were centrifuged at 5,000 g at 20 °C for 10 minutes. The cell pellets were washed with 3 mL of Tris-HCl 50 mM pH 7.5 buffer. One mL of lysis solution (7 M urea, 2 M thiourea, and 4% CHAPS), 50 mM DTT, and 5 μL mL^−1^ of protease inhibitor cocktail (Roche) were added to the sample. The cells were lysed in a sonicator during six 10-second cycles at 60% power, with 10-second intervals between cycles. After 60 minutes on ice, the samples were centrifuged for 60 minutes at 21,000 g and 4 °C, and the supernatant containing the solubilized proteins was retrieved and stored at −70 °C until analysis. The proteins obtained with this procedure were quantified using a 2D Quant kit (GE Healthcare) in an Ultrospec 5300 *pro* spectrophotometer (Amersham Biosciences), according to the manufacturer's protocol.

### 3.4. Two-Dimensional Gel Electrophoresis

A total of 450 μg of proteins from each sample were precipitated with methanol/chloroform and resuspended in IEF buffer (7 M urea, 2 M thiourea, 2% CHAPS, 0.002% bromophenol blue, 0.5% IPG buffer, and 50 mM DTT) and then applied to 24 cm IPG strips with pH 3-11 NL (GE Healthcare). The strips were rehydrated with each sample for 16 h at room temperature. Isoelectric focusing was conducted for six cycles in an Ettan ™ IPGphor II instrument (Amersham Biosciences): the first step was run at 100 V for 1 h, then 500 V for 2 h, a gradient to 1,000 V for 2 h, a gradient to 10,000 V for 3 h, followed by a gradient reaching 80,000 Vh and 75 μA/strip. Following focalization, the gel strips were equilibrated with solution containing 6 M urea, 30% glycerol, 75 mM of Tris-HCl pH 8.8, 2% SDS, and 0.002% bromophenol blue in two 15-minute steps at room temperature, the first with 64.8 mM DTT, and the second with 135.2 mM iodoacetamide. The second dimension was run in a 15% polyacrylamide gel in a vertical Ettan ™ DALTsix system (Amersham Biosciences) at 5 W per gel for 30 minutes, and then 17 W per gel, until the bromophenol blue reached the bottom of the gel. The gels were stained with Colloidal Coomassie Blue and a comparative analysis was made, with three biological replicates for each condition (control and exposed).

### 3.5. Image Acquisition and Quantitative Analysis of Protein Expression

The gels were scanned in an Image Scanner II (GE Healthcare) using the red filter, with an image resolution of 300 dpi. The gel images were analyzed with the software Image Master 2D Platinum v.7.0 (GE Healthcare) [42]. The spot volume was normalized considering the total volume of all spots detected in each gel. Each spot was compared between the different replicates; a spot was considered differentially expressed when there was a difference of at least 1.3-fold in the relative volume. These differential spot volumes were compared using ANOVA with a significance value of *p* < 0.05. The exclusive spots of one of the conditions were only taken into account when detected automatically in all replicates of the condition with a *p* < 0.05.

### 3.6. Protein Identification by MALDI-TOF/TOF

The differential spots were excised from the gels using an Ettan ™ Spot Picker (GE Healthcare). They were then dehydrated with acetonitrile and incubated on ice with trypsin (20 ng μL^−1^) (Promega Biosciences) for 1 h. The excess trypsin was removed, and the spots were digested at 58 °C for 30 minutes. The digested peptides were extracted in two 10-minute sonication cycles in 30 μL of a 5% formic acid/50% acetonitrile solution. The solution containing the peptides was concentrated in a SpeedVac to a volume of approximately 10 μL and then eluted and desalted with ZipTip (Millipore). The sample was then mixed 1:1 with a α-cyano-4-hydroxycinnamic acid (Sigma) matrix in a final volume of 1 μL and transferred to an Anchorchip 600 plate of a MALDI-TOF/TOF AutoflexIII ™ (Bruker Daltonics). This instrument was calibrated with Peptide Calibration Standard II (Bruker Daltonics), and the analysis was carried out in the positive/reflectron mode, controlled by FlexControl ™ software. The mass spectrograph spectra were analyzed using the MASCOT search program (Matrix Science) [43] and compared with the genomic data available for Proteobacteria deposited in the National Center for Biotechnology Information (NCBI).

### 3.7. Bioinformatics Tools

The sequences of the identified proteins were obtained from the NCBI database and classified using the COGnitor tool [44]. The online programs PSORTb v.3.0 [23] and PSLpred [22] were used to predict the location of each protein in the cell. *In silico* prediction of the proteome was made with JVirGel v.2.0 [21].

## 4. Conclusions

A large number of studies have focused on potential use of bacteria for eliminating pollutants. The ability of *C. violaceum* to eliminate cyanide has been described [45], but there is little information on the effect of this bacterium on chemical derivatives of cyanide, such as cyanate. Biological treatment of cyanate can be a less expensive alternative than the chemical and physical methods currently employed by the mining industry [46].

*Chromobacterium violaceum* is a free-living bacterium that we found resistant to concentrations of up to 1 mM of cyanate. We elucidated the biochemical behavior of this bacterium when it is exposed to cyanate. Cyanate induces expression of enzymes that combat oxidative stress, represses enzymes of the citric acid cycle, modifying the energy metabolism of the cell, and it represses enzymes involved in the degradation of amino acids, since cyanate is a rich source of nitrogen. We conclude that metabolization of cyanate by *C. violaceum* involves biological routes other than those controlled by the *cynRST* operon.

## Supplementary Files

Supplementary File 1 ZIP-Document (ZIP, 848 KB)

## References

[1] Hungria M., Astolfi-Filho S., Chueire L.M.O., Nicolás M.F., Santos E.B.P., Bulbol M.R., Souza-Filho A., Nogueira-Assunção E., Germano M.G., Vasconcelos A.R.T. (2005). Genetic characterization of *Chromobacterium* isolates from Black water environments in the Brazilian Amazon. Lett. Appl. Microbiol..

[2] Dall'Agnol L.T., Martins R.N., Vallinoto A.C.R., Ribeiro K.S.T. (2008). Diversity of *Chromobacterium violaceum* isolates from aquatic environments of state of Pará, Brazilian Amazon. Mem. Inst. Oswaldo Cruz.

[3] Lima-Bittencourt C.I., Astolfi-Filho S., Chartone-Souza E., Santos F.R., Nascimento A.M.A. (2007). Analysis of *Chromobacterium* sp. natural isolates from different Brazilian ecosystems. BMC Microbiol..

[4] Hungria M., Nicolás M.F., Guimarães C.T., Vasconcelos A.T.R. (2004). Tolerance to stress and environmental adaptability of *Chromobacterium violaceum*. Genet. Mol. Res..

[5] Brazilian Genome Consortium (2003). The complete genome sequence of *Chromobacterium violaceum* reveals remarkable and exploitable bacterial adaptability. Proc. Natl. Acad. Sci. USA.

[6] Carepo M.S.P., Azevedo J.S.N., Porto J.I.R., Bentes-Souza A.R., Batista J.S., Silva A.L.C., Schneider M.P.C. (2004). Identification of *Chromobacterium violaceum* genes with potential biotechnological application in environmental detoxification. Genet. Mol. Res..

[7] Lamblin A.F., Fuchs J.A. (1994). Functional analysis of the *Escherichia coli* K-12 cyn operon transcriptional regulation. J. Bacteriol..

[8] Walker J., Hambly F.J. (1895). Transformation of ammonium cyanate into urea. J. Chem. Soc..

[9] Baxter J., Cummings S.P. (2006). The current and future applications of microorganism in the bioremediation of cyanide contamination. Antonie Van Leeuwenhoek.

[10] Stark G.R. (1965). Reactions of cyanate with functional groups of proteins. III. Reactions with amino and carboxyl groups. Biochemistry.

[11] Srivastava V.K., Varshney N., Jaiswal A. (1993). *In vivo* effect of cyanate on serum and eye lens in rat. Indian J. Exp. Biol..

[12] Kamennaya N.A., Chernihovsky M., Post A.F. (2008). The cyanate utilization capacity of marine unicellular Cyanobacteria. Limnol. Oceanogr..

[13] Luque-Almagro V.M., Huertas M.J., Saéz L.P., Luque-Romero M.M., Moreno-Vivián C., Castillo F., Roldán M.D., Blasco R. (2008). Characterization of the *Pseudomonas pseudoalcaligenes* CECT5344 cyanase, an enzyme that is not essential for cyanide assimilation. Appl. Environ. Microbiol..

[14] Espie G.S., Jalali F., Tong T., Zacal N.J., So A.K.C. (2007). Involvement of the *cynABDS* operon and the CO_2_-concentrating mechanism in the light-dependent transport and metabolism of cyanate by Cyanobacteria. J. Bacteriol..

[15] Miyamoto K., Kosakai K., Ikebayashi S., Tsuchiya T., Yamamoto S., Tsujibo H. (2009). Proteomic analysis of *Vibrio vulnificus* M2799 grown under iron-repleted and iron-depleted conditions. Microb. Pathog..

[16] Roma-Rodrigues C., Santos P.M., Benndorf D., Rapp E., Sá-Correia I. (2010). Response of *Pseudomonas putida* KT2440 to phenol at the level of membrane proteome. J. Proteomics.

[17] Vintila S., Jonasson S., Wadensten H., Nilsson A., Andrén P.E., El-Shehawy R. (2010). Proteomic profiling of the Baltic Sea cyanobacterium *Nodularia spumigena* strain AV1 during ammonium supplementation. J. Proteomics.

[18] Cacace G., Mazzeo M.F., Sorrentino A., Spada V., Malorni A., Siciliano R.A. (2010). Proteomics for elucidation of cold adaptation mechanisms in *Listeria monocytogenes*. J. Proteomics.

[19] Boening D.W., Chew C.M. (1999). A critical review: General toxicity and environmental fate of three aqueous cyanide ions associated ligands. Water Air Soil Pollut..

[20] Guilloton M., Karst F. (1987). Cyanate specifically inhibits arginine biosynthesis in *Escherichia coli* K12: A case of by-product inhibition?. J. Gen. MIcrobiol..

[21] Kunz D.A., Nagappan O. (1989). Cyanase-mediated utilization of cyanate in *Pseudomonas fluorescens* NCIB 11764. Appl. Environ. Microbiol..

[22] Bhasin M., Garg A., Raghava G.P.S. (2005). PSLpred: Prediction of subcellular localization of bacterial proteins. Bioinformatics.

[23] Yu N.Y., Wagner J.R., Laird M.R., Melli G., Rey S., Lo R., Dao P., Sahinalp S.C., Ester M., Foster L.J. (2010). PSORTb 3.0: Improved protein subcellular localization prediction with refined localization subcategories and predictive capabilities for all prokaryotes. Bioinformatics.

[24] Hiller K., Grote A., Maneck M., Münch R., Jahn D. (2006). JVirGel 2.0: Computational prediction of proteomes separated via two-dimensional gel electrophoresis under consideration of membrane and secreted proteins. Bioinformatics.

[25] Castellana N., Bafna V. (2010). Proteogenomics to discover the full coding content of genomes: A computational perspective. J. Proteomics.

[26] Batista J.S.S., Torres A.R., Hungria M. (2010). Towards a two-dimensional proteomic reference map of *Bradyrhizobium japonicum* CPAC 15: Spotlighting “hypothetical proteins”. Proteomics.

[27] Quevillon E., Silventoinen V., Pillai S., Harte N., Mulder N., Apweiler R., Lopez R. (2005). InterProScan: Proteins domain identifier. Nucleic Acids Res..

[28] Poulsen P., Jensen K.F. (1991). Three genes preceding pyrE on the *Escherichia coli* chromosome are essential for survival and normal cell morphology in stationary culture and at high temperature. Res. Micrbiol..

[29] Ksiąźek K. (2010). Bacterial aging: From mechanistic basis to evolutionary perspective. Cell. Mol. Life Sci..

[30] Srivastava A.K., Bhargava P., Thapar R., Rai L.C. (2009). Differential response of antioxidative defense system of *Anabaena doliolum* under arsenite and arsenate stress. J. Basic Microbiol..

[31] Dukan S., Nyström T. (1998). Bacterial senescence: Stasis results in increased and differential oxidation of cytoplasmic proteins leading to developmental induction of the heat shock regulon. Genes Dev..

[32] Michaels R., Corpe W.A. (1965). Cyanide formation by Chromobacterium violaceum. J. Bacteriol..

[33] Rodgers P.B., Knowles C.J. (1978). Cyanide production and degradation during growth of *Chromobacterium violaceum*. J. Gen. Microbiol..

[34] Petrak J., Ivanek R., Toman O., Cmejla R., Cmejlova J., Vyoral D., Zivny J., Vulpe C.D. (2008). Déjà vu in proteomics. A hit parade of repeatedly indentified differentially expressed proteins. Proteomics.

[35] Wang P., Bouwman F.G., Mariman E.C.M. (2009). Generally detected proteins in comparative proteomics—A matter of cellular stress response?. Proteomics.

[36] Hansen H.M., Lehnherr H., Wang X., Mobley V., Jin D.J. (2003). *Escherichia coli* SspA is a transcription activator for bacteriophage P1 late genes. Mol. Microbiol..

[37] Williams M.D., Ouyang T.X., Flickinger M.C. (1994). Starvation-induced expression of SspA and SspB: The effects of a null mutation in SspA on *Escherichia coli* protein synthesis and survival during growth and prolonged starvation. Mol. Microbiol..

[38] Durmowicz M.C., Maier R.J. (1997). Roles of HoxX and HoxA in biosynthesis of hydrogenase in *Bradyrhizobium japonicum*. J. Bacteriol..

[39] Buhrke T., Friedrich B. (1998). hoxX (hypX) is a functional member of the *Alcaligenes eutrophus* hyp gene cluster. Arch. Microbiol..

[40] Szklarczyk D., Franceschini A., Kuhn M., Simonovic M., Roth A., Minguez P., Doerks T., Stark M., Muller J., Bork P. (2011). The STRING database in 2011: Functional interaction networks of proteins, globally integrated and scored. Nucleic Acids Res..

[41] Creczynski-Pasa T.B., Antônio R.V. (2004). Energetic metabolism of *Chromobacterium violaceum*. Genet. Mol. Res..

[42] Dowsey A.W., English J.A., Lisacek F., Morris J.S., Yang G.Z., Dunn M.J. (2010). Image analysis tools and emerging algorithms for expression proteomics. Proteomics.

[43] Perkins D.N., Pappin D.J.C., Creasy D.M., Cottrell J.S. (1999). Probability-based protein identification by searching sequence databases using mass spectrometry data. Electrophoresis.

[44] Tatusov R.L., Fedorova N.D., Jackson J.D., Jacobs A.R., Kiryutin B., Koonin E.V., Krylov D.M., Mazumder R., Mekhedov S.L., Nikolskaya A.N. (2003). The COG database: An updated version includes eukaryotes. BMC Bioinformatics.

[45] Farmazi M.A., Stagars M., Pensini E., Krebs W., Brandi H. (2004). Metal solubilization from metal-containing solid materials by cyanogenic *Chromobacterium violaceum*. J. Biotechnol..

[46] Akcil A. (2003). Destruction of cyanide in gold mill effluents: Biological *versus* chemical treatments. Biotechnol. Adv..

