# Supplementary figures and images for: Proteomics Analysis of the Effects of Cyanate on Chromobacterium violaceum Metabolism
Source: Genes (Basel). 2011 Oct 19;2(4):736–47. doi: 10.3390/genes2040736 (PMC3927592; doi:10.3390/genes2040736)

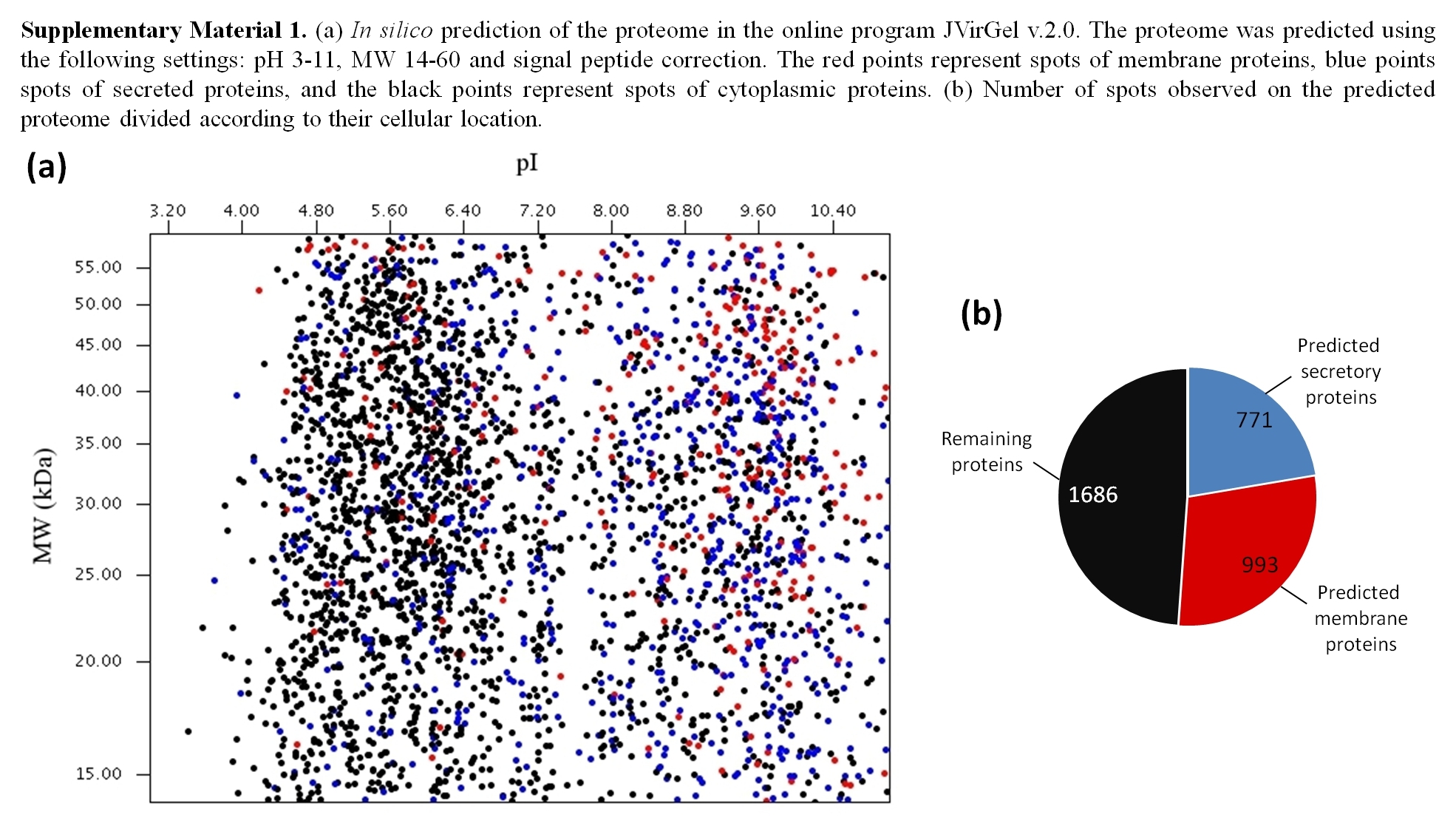

Supplement: Supplementary File 1 — ZIP-Document (ZIP, 848 KB) [file genes-02-00736-s001.zip › genes-10946-supplementary-final/supplementary material.jpg]
